# LINC00467 Promotes Tumor Progression *via* Regulation of the NF-kb Signal Axis in Bladder Cancer

**DOI:** 10.3389/fonc.2021.652206

**Published:** 2021-05-28

**Authors:** Jiawei Xiao, Lian Gong, Mengqing Xiao, Dong He, Liang Xiang, Zhanwang Wang, Yaxin Cheng, Liping Deng, Ke Cao

**Affiliations:** ^1^ Department of Oncology, Third Xiangya Hospital of Central South University, Changsha, China; ^2^ Department of Respiratory, The Second People’s Hospital of Hunan Province, Changsha, China

**Keywords:** LINC00467, NF-kb, proliferation, invasion, bladder cancer

## Abstract

**Purpose:**

Long non-coding RNAs (lncRNAs) play an important role in the occurrence and development of bladder cancer, but the underlying molecular mechanisms remain largely unknown. In this study, we found that LINC00467 was significantly highly expressed in bladder cancer through bioinformatic analysis. The present study aimed to explore the role of LINC00467 in bladder cancer and its possible underlying molecular mechanisms.

**Methods:**

The expression of LINC00467 was obtained from GEO (GSE31189), the TCGA database, and qRT-PCR. The role of LINC00467 in bladder cancer was assessed both *in vitro* and *in vivo*. RIP, RNA pulldown, and CO-IP were used to demonstrate the potential mechanism by which LINC00467 regulates the progression of bladder cancer.

**Results:**

Through the analysis of GEO (GSE133624) and the TCGA database, it was found that LINC00467 was highly expressed in bladder cancer tissues and that the expression of LINC00467 was significantly negatively correlated with patient prognosis. Cell and animal experiments suggest that LINC00467 promotes the proliferation and invasion of bladder cancer cells. On the one hand, LINC00467 can directly bind to NF-kb-p65 mRNA to stabilize its expression. On the other hand, LINC00467 can directly bind to NF-kb-p65 to promote its translocation into the nucleus to activate the NF-κB signaling pathway, which promotes the progression of bladder cancer.

**Conclusions:**

LINC00467 is highly expressed in bladder cancer and can promote the progression of bladder cancer by regulating the NF-κB signaling pathway. Therefore, targeting LINC00467 is very likely to provide a new strategy for the treatment of bladder cancer and for improving patient prognosis.

## Introduction

Bladder cancer is one of the most common tumors of the genitourinary system, and it mainly occurs in men. There are approximately 549,000 new cases and 200,000 deaths worldwide every year (1). According to its clinical characteristics, bladder cancer can be divided into non-muscle-invasive bladder cancer (NMIBC) and muscle-invasive bladder cancer (MIBC). NMIBC is mainly treated by transurethral resection, intravesical BCG infusion, or bladder infusion chemotherapy. Due to its rapid progression and high recurrence rate, MIBC is mainly treated with radical cystectomy and neoadjuvant chemotherapy (2). Therefore, exploring new molecular markers and therapeutic targets for improving the clinical efficacy of treatment in patients with bladder cancer is important.

Nuclear factor-kb (NF-kb) is a family of transcription factors composed of five different DNA-binding proteins: P50, P52, P65, RELB, and c-Rel (3). In the resting state, they usually form homodimers or heterodimers in the cytoplasm and bind to the IkB protein (inhibitor of NF-κB). Activation of the NF-κB signaling pathway depends on the activation of IκB kinases (IKK). The activation of IKK complexes phosphorylates IkB proteins, triggering their degradation via the proteasome (3). The influence of NF-κB on tumor progression is mainly related to its classical pathway. In the classical pathway of NF-κB, tumor necrosis factor (TNF), IL-1, lipopolysaccharide (LPS), and other substances activate IKK. Activated IKK can induce phosphorylation of IkB protein and depolymerize it from the P50/P65 complex. The released P50/P65 complex is further activated after various post-translational modifications, translocates to the nucleus, binds to its target genes, and promotes the transcription of target genes that regulate cell proliferation, apoptosis, metastasis, invasion, and other biological processes (4, 5). Therefore, the NF-κB signaling pathway is closely related to the occurrence and development of tumors.

In recent years, many studies have found that long non-coding RNAs (lncRNAs) play an important role in the progression of bladder cancer. Long non-coding RNAs (lncRNAs) are transcripts longer than 200 nucleotides that do not encode or encode short peptides. They can participate in the regulation of various physiological and pathological processes, such as cell proliferation, apoptosis, invasion, metastasis, and autophagy (6, 7). Studies have shown that lncRNAs can directly participate in the regulation of the NF-κB signaling pathway activation process, which affects the phenotype of tumor cells. For example, in breast cancer, the lncRNA NKILA induced by the NF-κB signaling pathway can bind to the NF-kb/IkB complex and mask the phosphorylation site of IkB, which inhibits IKK-induced phosphorylation of IkB and activation of NF-κB, thereby inhibiting the metastasis of breast cancer cells (6). In addition, in prostate cancer, lncRNA DRAIC can bind to IKK and reduce its kinase activity on IkB, which inhibits the activation of NF-κB, thereby inhibiting the proliferation and metastasis of prostate cancer cells (8).

In this study, we analyzed the GEO and TCGA databases and found that LINC00467 was significantly highly expressed in bladder cancer tissue and that the expression of LINC00467 was significantly negatively correlated with the patient’s disease-free survival, which indicates that LINC00467 may be closely related to the recurrence and metastasis of bladder cancer patients. Further studies have shown that LINC00467 can not only directly bind NF-kb-p65 mRNA to stabilize its expression, but can also directly bind to NF-kb-p65 to promote nuclear translocation of NF-kb-p65. This activates the NFKB signaling pathway, thereby promoting tumor cell proliferation and invasion. Our findings are very likely to provide new targets and strategies for the treatment of bladder cancer.

## Materials and Methods

### Bioinformatics Analysis

The expression profiles of lncRNAs in human bladder cancer tissues were collected from GEO (GSE133624), TANRIC (http://bioinformatics.mdanderson.org/main/TAN RIC : Overview) (9) and GEPIA (http://gepia.cancer-pku.cn/) (10) which was used for patient survival analysis and correlation prediction. catRAPID (http://service.Tartaglialab.com/page/catrapid_group) (11) was used to predict RNA-protein binding. IntaRNA2.0 (http://rna.informatik.uni-freiburg.de/IntaRNA/Input.jsp) (12) was used to predict lncRNA-mRNA binding.

### Tissues and Cell Lines

All human BUC specimens were collected from patients at Third Xiangya Hospital (2016-2018), and all patients were diagnosed by histopathology and radical cystectomy. These clinical specimens were previously approved by the patient and approved by the ethics committee for research purposes. Six pairs of BUC tissue samples and corresponding adjacent non-tumor tissue samples were stored at -80°C immediately after radical cystectomy and used for qRT-PCR and/or Western blotting. Human bladder cancer T24 and RT4 cells were purchased from ATCC (Rockville, MD, USA). The cells were cultured in DMEM (Invitrogen) bovine serum (FBS) (Gibco) in a humidified atmosphere at 37°C with 5% CO2 supplemented with 10% fetal.

### Cell Transfection

siRNA, overexpression plasmid of LINC00467, and negative controls were purchased from RiboBio (Guangzhou, China). Lipofectamine 3000 (Invitrogen, Carlsbad, CA, USA) was used following the instructions for plasmid transfection as previously described (13). Transfection efficiency was determined **Supplementary Files 2A–C**. The LINC00467 siRNA were purchased from Ribobio (Guangzhou, China). The following siRNA sequences were used: Forward 5-GAUGCUCUGUAAACCACAUTT-3; Reverse 5-AUGUGGUUUACAGAGCAUCTT-3.

### Quantitative Real Time PCR

Total RNA was extracted from the cells using TRIzol (Invitrogen), and the purity of the RNA was assessed spectrophotometrically (A260/A280>1.8). Approximately 1μg of total RNA was reverse transcribed into cDNA using an MMLV reverse transcriptase reagent (Promega, Madison, WI, USA). According to the manufacturer’s instructions, real-time qRT-PCR was used to detect LINC00467 and NF-kb-p65 mRNA expression using a SYBR Green PCR Master Mix on an ABI 7500 sequence detection system (Life Technologies). All experiments were performed in triplicate using β-actin or U1 as internal controls. Relative expression levels were calculated using the 2^-ΔΔCt^ method. The following primer sequences were used: LINC00467-F: 5-TCGTCTTCAGGAAGCCAGAC-3; R: 5- TGGAAATCAAAAGGGTCAGC-3; NF-kb-p65 -F: 5-ATGTGGAGATCATTGAGCAGC-3; R: 5-CCTGGTCCTGTGTAGCCATT-3; β-actin -F: 5-CATGTACGTTGCTA TCCAGGC-3; R: 5-CTCCTTAATGTCACGCACGAT-3; U1 -F: 5- GGGAGATACCATGATCACGAAGGT-3; R: 5- CCACAAATTATGCAGTCGAGTTTCCC-3.

### Wound-Healing Assays

Approximately 5 × 10^5^ cells/mL were plated in 6-well plates, and the plates were observed for monolayers that had covered >90% of the bottom of the well or had covered it completely on the second day after plating. Next, a 10-μL pipette tip was used to scratch a straight mark down the middle of the monolayer. After creating the wound, the 10% FBS–containing medium was replaced with medium containing a 1% FBS concentration, and photos taken at 0 h were used as the control. The plates were then placed in a 5% CO_2_ incubator at 37°C, and photos were captured after 48 h.

### MTT Assays

Exponentially growing cells were inoculated in 96-well plates at 1 × 10^4^ cells per well (100 μL medium) and incubated for 24 h (37°C, 5% CO_2_). MTT (50 μl of MTT (Sigma Chemicals, St. Louis, MO, USA; 5 mg/ml in PBS) was then added to each well, and the cells were cultured for an additional 4 h. Subsequently, the supernatant was aspirated, and 150 μl of DMSO was added to each well to dissolve the crystals. Cell proliferation was estimated using a microplate reader at a wavelength of 570 nm.

### Clone Formation Assays

The monolayer culture cells in the logarithmic growth phase were trypsinized and counted, and 1000 cells per well were inoculated into culture dishes (60 mm) and kept at 37°C with 5% CO_2_ and saturated humidity for 2–3 weeks. The cells were fixed with 1 ml of paraformaldehyde for 30 min, and the fixed cells were stained with hematoxylin and counted under a microscope. The clone was counted if the cell number of the clone was increased to 50.

### Transwell Assays

After transfection for 24 h, bladder cancer cells were collected and washed with PBS as well as serum-free DMEM. The upper chamber of the Transwell was pre-coated with Matrigel (0.2μg/μl, diluted in DMEM medium, Corning, USA), and 600 μl DMEM containing 20% FBS was added to the lower transwell chamber. A 200 μl (5×10^4^ cells/mL) cell suspension of DMEN without FBS was plated into the upper chamber. The cells were incubated at 37°C for 48 h. Cells that had invaded across the membrane were fixed with 75% methanol for 30 min and stained with 0.5% crystal violet for 60 min. Stained cells were counted in 10 random microscopic fields per membrane (microscope: DMB5-2231P1, DMB HK Ltd., Hong Kong, China).

### Western Blotting

Western blotting was performed as described previously (14, 15). Tissues and cells were washed with PBS and then lysed on ice for 30 min with RIPA lysis buffer containing 10% protease inhibitor cocktail (Roche), and protein concentration was determined using a BCA Protein Assay Kit (Thermo Scientific). Subsequently, the cell lysate containing 20-40 μg of protein was separated with a 10% SDS-polyacrylamide gel and transferred to a PVDF membrane, and the membrane was blocked in 5% skim milk powder (formed in PBS) and incubated with anti-NF-kb-p65 (Abcam/ab16502;1:1000), anti-IKBαb (Cst/#4812;1:1000), anti-IKBβ (Abcam/ab7547;1:1000), anti-Histone H3 (CST,#4499;1:1000), and anti-β-actin (Ptgcn/66009-1-Ig;1:2000) overnight at 4°C followed by addition of secondary antibody. The secondary antibody was discarded, and the band was imaged *via* ECL and quantified using the optical density analysis software Quantity One (Bio-Rad). GAPDH was used as an internal reference to standardize the expression of other proteins.

### Xenograft Mouse Model

Female nude mice (5-week-old, 17.9 ± 0.82 g) were purchased from Shanghai Laboratory Animal Center (SLAC, Shanghai, China). All animal experimental procedures were performed in accordance with the guidelines defined by the ethics committee and ethics committee approval. The RT4 cells were transfected with siRNA targeting LINC00467, overexpression plasmids, or vector plasmids. Stably transfected bladder cancer cells were collected during logarithmic growth phase. Then, the bladder cancer cells were washed and resuspended in PBS to achieve a cell concentration of 1 × 10^7^ cells/ml. A 200 μL cell suspension was subcutaneously implanted into the right armpit of nude mice (the nude mice were divided randomly into three groups of 12), and macroscopic tumors were grown in the mice. The size of the tumor was calculated by measuring its length (L) and width (W) every 3 days. The nude mice were sacrificed after 25 days, and tumor volumes were measured according to the formula V = 1/2 (L × W^2^). All experiments were approved by the Ethics Committee of the Third Xiangya Hospital of Central South University.

### Immunohistochemistry

After paraffin-embedded tissue was sliced, dewaxed, hydrated, and antigen-repaired, with additional blockage of endogenous peroxidase, anti-Ki67 (Genetex, GTX 16667) was added, and the samples were refrigerated at 4°C overnight. Polymer enhancer was added dropwise for 20 min at room temperature, and a biotin-labeled secondary antibody was added dropwise. The samples were then incubated at room temperature for 30 min. DAB was used as the color body while hematoxylin was used as the color former. Finally, a conventional dehydrated and transparent neutral gum seal was used. The degree of immunostaining of the paraffin-embedded sections was assessed and scored by two independent pathologists who were blinded to the histopathological features and patient data. The score was determined by combining the proportion of positively stained tumor cells and staining intensity. Specific scoring rules were based on previous research.

### Fluorescence *In Situ* Hybridization

Fluorescence *in situ* hybridization (FISH) was performed to detect the localization and expression of LINC00467 in BUC cells. The blue DAPI and red FISH probes were purchased from RiboBio (Guangzhou, China). The subcellular localization of LINC00467 was detected using a FISH kit (RiboBio, Guangzhou, China) according to the manufacturer’s instructions.

### Immunoprecipitation (IP)

The RIP assay was performed using an EZ-Magna RIP kit (Millipore, MA, USA). Briefly, 1×10^7^ cells were harvested and lysed with RIP lysis buffer with one freeze-thaw cycle. The cell extract was co-immunoprecipitated using anti-HuR (Proteintech, 11910-1-AP), and the retrieved RNA was subjected to qRT-PCR analysis. Rabbit IgG was used as the NC, and purified RNA was then analyzed by qRT-PCR using RIP Primers specific for the U1 snRNA.

### CO-IP

CO-IP was performed as described previously (16). Adherent cells were taken from the 10 cm cell culture dish, the cell culture medium was aspirated and washed once with PBS, the cells were lysed with 1 ml IP buffer and mixed with 2 μg anti-NF-kb**-**p65 at 4°C, and then incubated overnight for rotation. Twenty µL of fully resuspended, Protein A+G Agarose was added, and the sample was shaken slowly for 2 h at 4°C. After centrifugation at 2500 rpm for 5 min, the supernatant was carefully aspirated and the IP complex was placed 5 times in IP buffer as well as 20 µl of 1X SDS-PAGE electrophoresis loading buffer Vortex. The pellet was resuspended and the sample from the bottom of the tube was centrifugated by instantaneous high-speed centrifugation.

### Stability and α-Amanitin Treatment

We inoculated LINC00467 siRNA, siRNA NC, and LINC00467 and T24/RT4 cells overexpressing the blank plasmid into 6-well plates. Cells were treated with 50 μg/ml of α-amanita toxin after 24 h, and cells were harvested for RNA purification and RT-PCR after 6, 12, 18, 24, and 48 h of treatment. We collected three independent samples for each data point, and all samples had untreated and untransfected matched samples for RNA purification and data analysis.

### Statistical Analysis

GraphPad Prism 8.02 was used for all statistical analyses, and all experiments were repeated three times. Data are expressed as mean ± SD. Significant differences between the two groups were performed using Student’s t-tests, while significant differences between groups were analyzed using a one-way ANOVA followed by a Dunnett’s test. A p value <0.05 was considered to be a significant difference.

## Results

### LINC00467 Is Highly Expressed in Bladder Cancer and Is Correlated With Poor Prognosis

**Figure 1:**
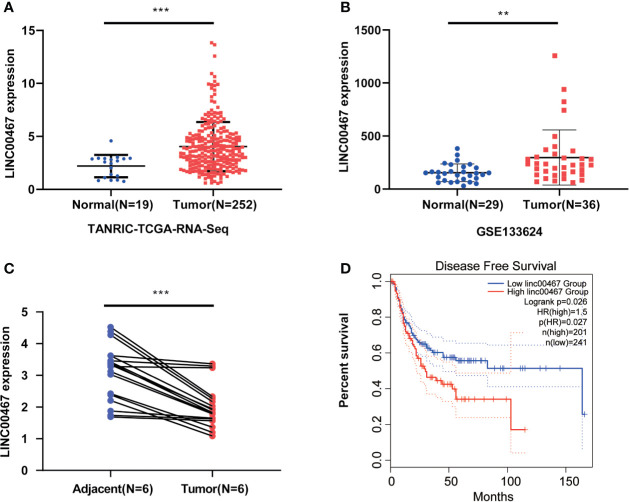
LIN00467 is highly expressed in bladder cancer and is negatively correlated with prognosis. **(A, B)** TCGA database and GSE133624 showed LINC00467 expression is increased in bladder cancer tissues compared with paired adjacent normal tissues. **(C)** The q-PCR method was used to detect the expression of LINC00467 in 6 pairs of bladder cancer tissues and paired adjacent normal tissues. **(D)** The GEPIA database showed that LINC00467 was negatively correlated with the disease-free survival of patients with bladder cancer **P < 0.01, ***P < 0.001.

TANRIC (n=271) and GSE133624 (n=55) suggest that LINC00467 expression is increased in bladder cancer tissues compared with paired adjacent normal tissues (**Figures 1A, B**). To verify the results of bioinformatics analysis, we detected the expression of LINC00467 in six pairs of bladder cancer tissues and paired adjacent normal tissues, which confirmed that LINC00467 was significantly upregulated in bladder cancer tissues (**Figure 1C**). Further analysis of the GEPIA database showed that LINC00467 expression was negatively correlated with the disease-free survival of patients with bladder cancer (**Figure 1D**).

### LINC00467 Promotes Proliferation and Invasion in Bladder Cancer

**Figure 2:**
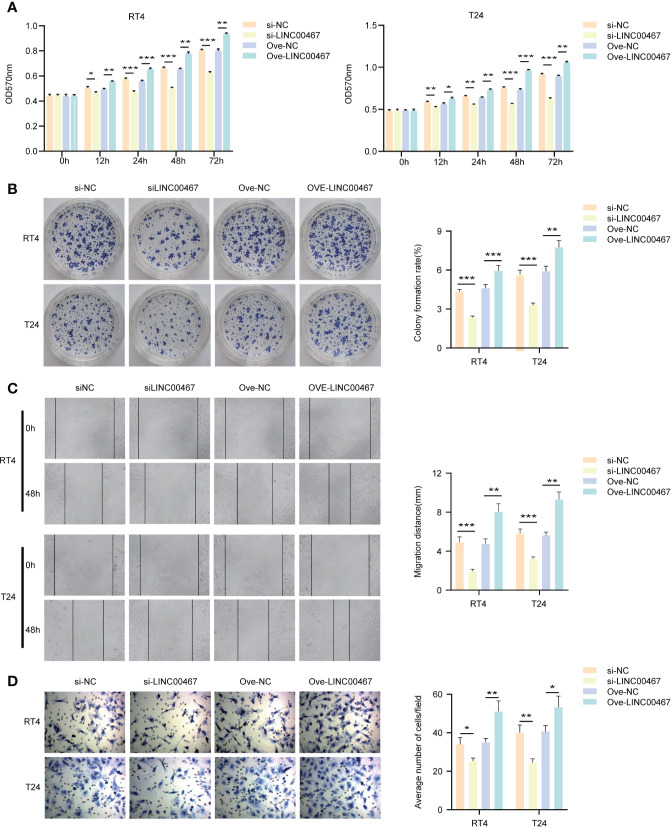
LINC00467 can promote the proliferation and invasion of bladder cancer *in vitro*. **(A, B)** MTT and cell clone formation experiments respectively detect the proliferation ability. **(C, D)** Wound-healing assays (×200) and transwell experiments (×200) respectively detect the migration and invasion ability. *P < 0.05, **P < 0.01, ***P < 0.001.

To explore the biological functions of LINC00467 in bladder cancer cells, T24 and RT4 cell lines were used to knockdown or overexpress LINC00467. We found that LINC00467 promoted the proliferation of bladder cancer cells using the MTT assay and colony forming assay (**Figures 2A, B**) and showed that LINC00467 accelerated the migration and invasion of bladder cancer cells using migration and invasion assays (**Figures 2C, D**).

### LINC00467 Can Bind to NF-kb-p65 mRNA and Increase Its Stability

**Figure 3:**
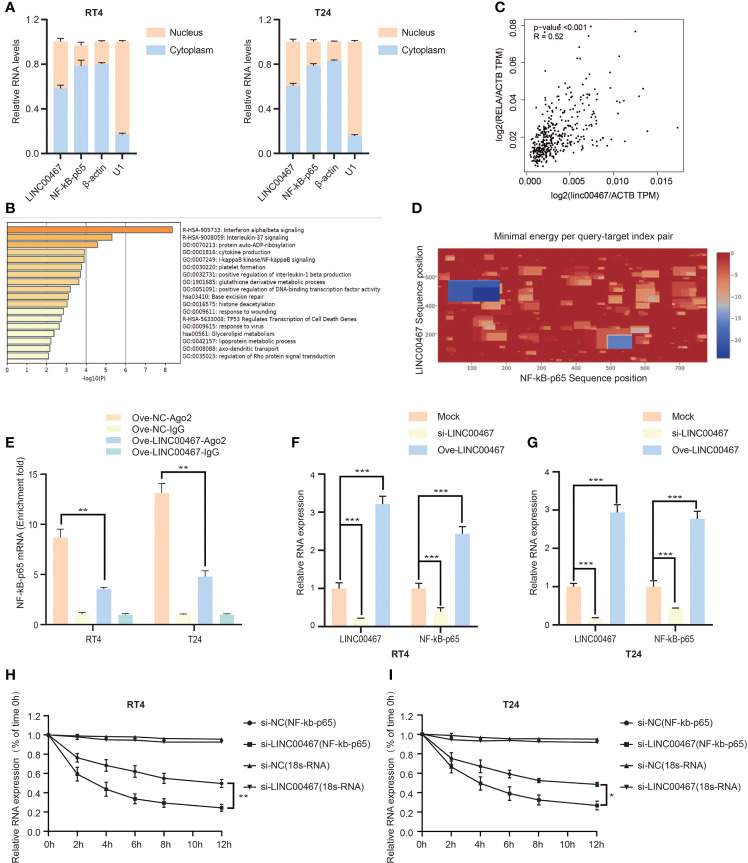
LINC00467 can bind to NF-kb-p65 mRNA and increase its stability. **(A)** The cytoplasm and nuclear RNA of RT4/T24 cells were extracted, and the expression of LINC00467 and NF-kb-p65 mRNA was detected by q-PCR. **(B)** The metascape website shows that LINC00467 interacts with the NF-κB signaling pathway. **(C)** The GEPIA website showed that LINC00467 and NF-kb-p65 mRNA expression were positively correlated in bladder cancer. **(D)** The IntaRNA 2.0 website showed that there may be binding sites between LINC00467 and NF-kb-p65 mRNA. **(E)** RIP assay showed that LINC00467 binds to NF-kb-p65 mRNA. **(F, G)** q-PCR to detect NF-kb-p65 mRNA expression after knockdown or overexpression of LINC00467. **(H, I)** RNA stability experiments show that the stability of NF-κb-p65 mRNA decreases after knocking out LINC00467. *P < 0.05, **P < 0.01, ***P < 0.001.

To determine the molecular mechanism by which LINC00467 promotes bladder cancer cell proliferation and invasion, we analyzed its localization in cells and found that LINC00467 is located both inside the nucleus and the cytoplasm. (**Figure 3A**). Subsequently, we found the LINC00467 co-expressed mRNAs on the Co-LncRNA website and performed functional enrichment analysis using Metascape. The results showed that LINC00467 is very likely to interact with the NF-κB signaling pathway (**Figure 3B**). Therefore, we analyzed the correlation between LINC00467 and NF-kb-p65 mRNA through the GEPIA website and found that LINC00467 and NF-kb-p65 mRNA expression were positively correlated in bladder cancer (**Figure 3C**). Bioinformatics analysis showed that LINC00467 harbors the binding sequences for NF-kb-p65 mRNA on the IntaRNA 2.0 website (**Figure 3D**). To verify our hypothesis, we performed RIP experiments and showed that overexpression of LINC00467 reduced the binding of AGO2 to NF-kb-p65 mRNA, which indicated that LINC00467 is very likely to directly bind to NF-kb-p65 mRNA (**Figure 3E**). Therefore, we knocked out LINC00467 in the bladder cancer cell line T24/RT4 and found that the expression of NF-kb-p65 mRNA decreased, while the expression of NF-kb-p65 mRNA increased after overexpression of LINC00467 (**Figures 3F, G**). This indicates that LINC00467 is very likely to stabilize the expression of NF-kb-p65 mRNA. Therefore, we performed RNA stability experiments and showed that LINC00467 silencing markedly shortened the half-life of NF-kb-p65 mRNA, whereas LINC00467 overexpression markedly increased the half-life of NF-kb-p65 mRNA (**Figures 3H, I**). In summary, LINC00467 directly binds to NF-kb-p65 mRNA and increases its stability.

### LINC00467 Can Bind to NF-kb-p65 to Increase Its Translocation Into the Nucleus

**Figure 4:**
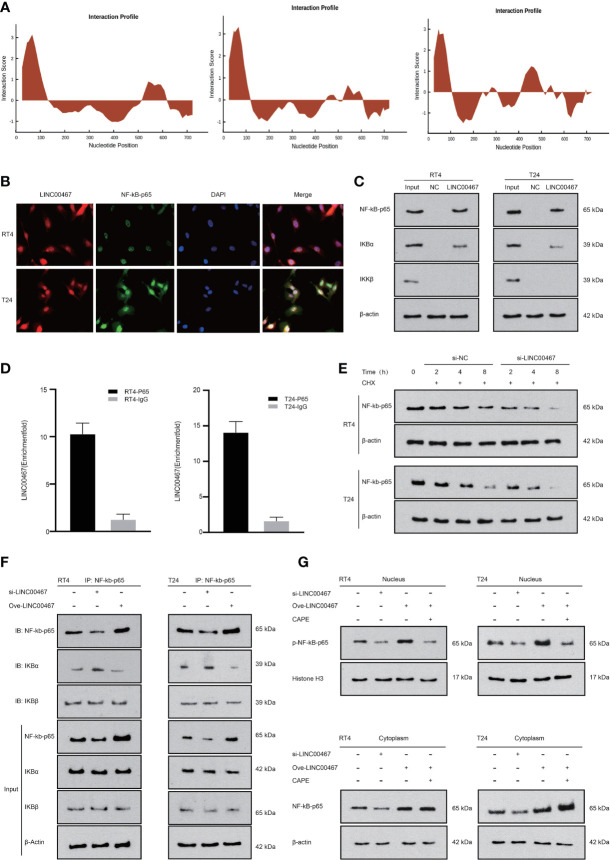
LINC00467 can bind to NF-kb-p65 to increase its stability and promote its translocation into the nucleus. **(A)** The catRAPID website shows that LINC00467 can bind to NF-kb-p65. **(B)** Fluorescence *in situ* hybridization results show that LINC00467 co-localizes with NF-kb-p65. **(C)** RNA pull-down experiments show that LINC00467 can bind to the NF-kb-p65/IKBα complex. **(D)** The RIP experiment confirmed that LINC00467 can bind to NF-kb-p65. **(E)** Protein stability experiments showed that LINC00467 knockdown decreased the protein stability of NF-kb-p65. **(F)** CO-IP method uses NF-kb-p65 as the precipitated antibody to precipitate the cell lysate to detect the expression levels of NF-kb-p65, IKBα, and IKBβ after overexpression and knockdown of LINC00467. **(G)** WB method detects the expression levels of nuclear p-NF-kb-p65 and cytoplasmic NF-kb-p65 after knockdown or overexpression of LINC00467.

To further study the mechanism of the interaction between LINC00467 and NF-kb-p65, we found that LINC00467 probably combined with NF-kb-p65 through the catRAPID website (**Figure 4A**). We found that LINC00467 and NF-kb-p65 colocalized in the cell through immunofluorescence colocalization experiments (**Figure 4B**). The binding relationship between LINC00467 and NF-kb-p65 was detected by both RNA pull-down experiments and RIP experiments (**Figures 4C, D**). To investigate whether LINC00467 affects the stability of NF-kb-p65, LINC00467 was knocked down and cycloheximide (CHX) was used to inhibit *de novo* protein synthesis in T24 and RT4 cells. Western blot analysis showed that the stability of NF-kb-p65 was decreased in the LINC00467 knockdown group compared to the NC group (**Figure 4E** and **Supplementary Material 1A**). In addition, we also found that LINC00467 overexpression increased NF-kb-p65 expression and decreased the binding of IKBα to NF-kb-p65 through COIP experiments. However, LINC00467 silencing had the opposite effect. Therefore, we propose that LINC00467 can dissociate IKBα from the NF-kb-p65/IKBα complex, after which NF-kb-p65 translocates from the cytoplasm into the nucleus to exert its function (**Figure 4F**). Therefore, we transfected si-LINC00467 or a LINC00467 overexpression plasmid into T24/RT4 cells to analyze the effect of LINC00467 on nuclear translocation of NF-kb-p65. We found that LINC00467 overexpression increased the expression of p-NF-kb-p65 in the nucleus and cytoplasm. The expression of NF-kb-p65 and p-NF-kb-p65 was significantly increased by LINC00467 overexpression and CAPE treatment effectively inhibited p-NF-kb-p65 expression in the nucleus, but it had no significant effect on the expression of NF-kb-p65, indicating that LINC00467 can increase the translocation of NF-kb-p65 into the nucleus (**Figure 4G** and **Supplementary Material 1B**). In summary, LIN00467 can bind to NF-kb-p65 and increase its translocation into the nucleus.

### LINC00467 Can Regulate the Proliferation and Invasion of Bladder Cancer Through the NF-kb Signaling Pathway

**Figure 5:**
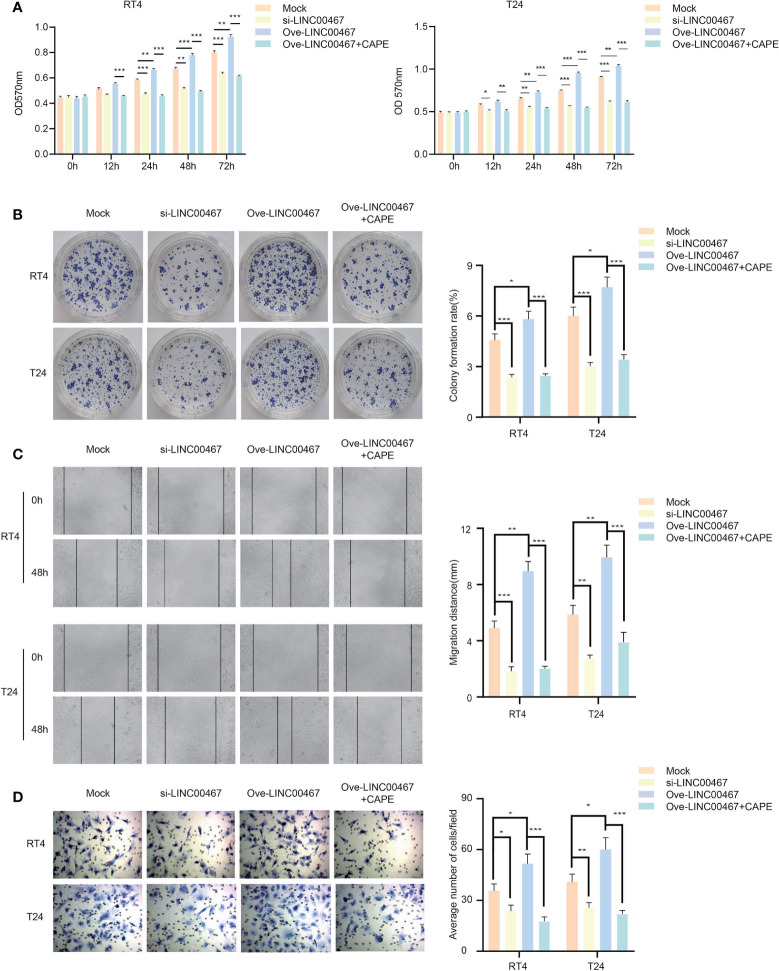
LINC00467 can regulate the proliferation and invasion of bladder cancer through the NF-kb signaling pathway. **(A, B)** MTT and cell clone formation experiments detected the proliferation ability. **(C, D)** Wound-healing assays(×200) and Transwell assays(×200) detected the ability of migration and invasion. *P < 0.05, **P < 0.01, ***P < 0.001.

The NF-κB signaling pathway can promote tumor proliferation and invasion in a variety of ways, and thus plays an important role in the occurrence and development of bladder cancer. LINC00467 can increase the stability of NF-kb-p65 and promote its translocation into the nucleus. Therefore, we propose that LINC00467 regulates the proliferation and invasion of bladder cancer through the NF-κB signaling pathway. Compared to the negative control group, in the LINC00467 overexpression + CAPE group, the proliferation ability of bladder cancer cells was markedly reduced; however, opposite results were obtained in the LINC00467 overexpression group (**Figures 5A, B**). Similarly, the results of both the wound-healing assays and transwell assays showed that bladder cancer cells in the LINC00467 overexpression + CAPE group had lower migration and invasion ability than the control group, while the migration and invasion of bladder cancer cells was accelerated in the LINC00467 overexpression group (**Figures 5C, D**). In summary, LINC00467 regulates the proliferation and invasion of bladder cancer through the NF-κB signaling pathway.

### LINC00467 Can Promote the Proliferation of Bladder Cancer *In Vivo*

**Figure 6:**
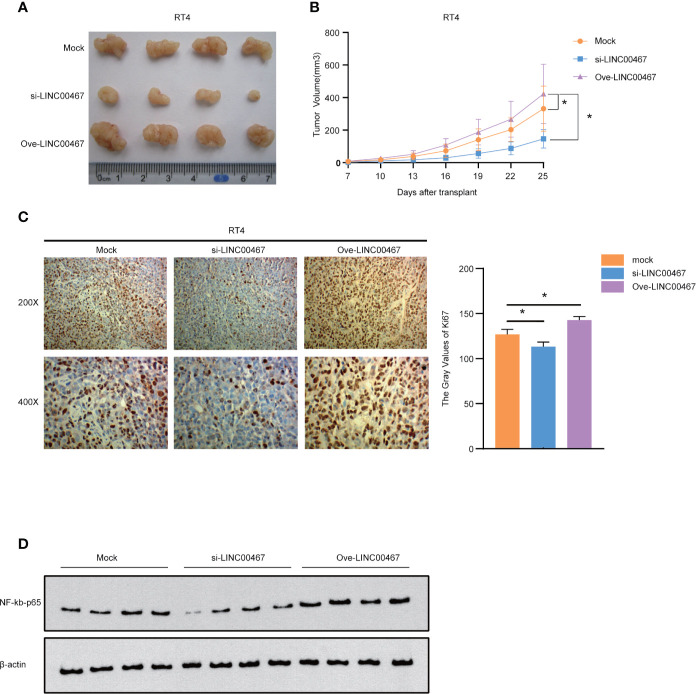
LINC00467 can promote the proliferation of bladder cancer *in vivo*. **(A, B)** Subcutaneous tumor formation experiments in nude mice showed that the LINC00467 knockdown group grew slower and smaller than those from control cells, while the opposite results were obtained in the LINC00467 overexpression group. **(C)** Immunohistochemical analysis of Ki67 protein expression **(D)** WB detects the expression levels of NF-kb-p65. *P < 0.05.

We further explored whether LINC00467 promoted the proliferation of bladder cancer cells *in vivo*. We found that subcutaneous tumors in nude mice formed from the LINC00467 knockdown group grew dramatically slower and smaller than those from control cells, while the opposite results were obtained in the LINC00467 overexpression group (**Figures 6A, B**). Immunohistochemistry (IHC) showed that tumor sections from LINC00467 overexpression mice exhibited strong Ki67 staining signals, whereas tumor sections displayed weak Ki67 expression in the LINC00467 knockdown group (**Figure 6C**). In addition, we detected the expression levels of NF-kb-p65 in tumor tissues from the mock group, LINC00467 knockdown group, and LINC00467 overexpression group. The results showed that the expression level of NF-kb-p65 was increased significantly in the LINC00467 overexpression group, while the LINC00467 knockdown group showed an opposite effect (**Figure 6D** and **Supplementary Material 1C**). In conclusion, our study demonstrated that LINC00467 promotes the proliferation of bladder cancer through the NF-κB signaling pathway.

## Discussion

In recent years, the role of non-coding RNAs in tumors has attracted increasing attention. Many studies have shown that lncRNAs play an important role in the occurrence and development of tumors. In our previous studies, we found that LINC00467 can promote the invasion and metastasis of lung adenocarcinoma (13), but its role in bladder cancer needs to be further elucidated. We first discovered that LINC00467 is highly expressed in bladder cancer using the TCGA and GEO databases. We found that LINC00467 plays a critical role in promoting the proliferation and invasion of bladder cancer *in vivo* and *in vitro*, suggesting that LINC00467 is likely to function as an oncogene in bladder cancer. These results were consistent with those of previous studies. Therefore, this study provides a critical new target for the treatment of bladder cancer. The mechanism by which lncRNAs affect physiological and pathological processes in organisms is very complicated. Currently, the widely recognized mechanism models of lncRNAs include signals, decoys, guides, and scaffolds (17). For example, in the process of DNA damage induction, the “signaling” lncRNAs, PANDA, are induced by P53 to interact with the transcription factor NF-YA to inhibit the expression of pro-apoptotic genes (18). The decoying lncRNAs affect the expression of their target genes by competitively binding to transcription factors and nuclear receptors, as well as their corresponding DNA sequences in the cell nucleus. For example, lncRNA Gas5 forms a motif that competitively binds to the DNA binding domain of the glucocorticoid receptor, thus inhibiting the expression of the glucocorticoid receptor (19). The decoying lncRNAs can also combine with miRNA through the mechanism of ceRNAs to affect the regulation of miRNAs on target genes (20). The “guiding” lncRNAs regulate transcription by binding to transcription-related proteins and transporting them to specific genomic DNA regions. For example, HOTAIR affects the metastasis and invasion of tumor cells by binding to PRC2 (21). The “scaffold” lncRNAs such as HOTAIR and ANRIL simultaneously bind to a variety of related regulatory proteins through different binding sites to form a complex, which can simultaneously affect downstream gene expression through multiple mechanisms (22–24). In summary, lncRNAs mainly function by interacting with various proteins. Studies have shown that lncRNAs can regulate the activation of the NF-κB signaling pathway by interacting with NF-kb-p65 (25). In this study, we found that there are binding sites between LINC00467 and NF-kb-p65 through catRAPID website analysis. The FISH experiment verified that LINC00467 and NF-kb-p65 were colocalized in the cell. Next, we performed RNA pull-down and RIP experiments, which showed that LINC00467 can bind to NF-kb-p65. To explore the relationship between LINC00467 and NF-kb-p65, we performed WB experiments, which showed that LINC00467 knockdown significantly reduced the expression of NF-kb-p65 while LINC00467 overexpression increased the expression of NF-kb-p65. Therefore, LINC00467 and NF-kb-p65 expression was positively correlated in bladder cancer. We know that the activation of the NF-kb signaling pathway often involves the nuclear translocation of NF-kb-p65, so we suspect that LINC00467 is very likely to promote the translocation of NF-kb-p65 into the nucleus by directly binding to NF-kb-p65, thereby activating the NF-κB signaling pathway. Therefore, we found that LINC00467 overexpression increased NF-kb-p65 expression and decreased the binding of IKBα to NF-kb-p65 through COIP experiments. However, LINC00467 silencing had the opposite effect. We overexpressed LINC00467 and then used CAPE to inhibit the activation of NF-kb-p65. The results showed that LINC00467 overexpression increased the expression of p-NF-kb-p65 in the nucleus. However, the expression levels of p-NF-kb-p65 decreased significantly when the NF-kb-p65 phosphorylation inhibitor CAPE was added. However, CAPE had no significant effect on the expression of NF-kb-p65 in the cytoplasm. In summary, our research shows that LINC00467 can directly bind to NF-kb-p65, promote the translocation of NF-kb-p65 into the nucleus, and activate the NF-κB signaling pathway.

Studies have found that lncRNAs can also play a role by directly binding to mRNA. Therefore, we analyzed the online data of GEPIA and IntaRNA 2.0, found that LINC00467 and NF-kb-p65 mRNA expression were positively correlated, and that LINC00467 and NF-kb-p65 mRNA were likely to have binding sites. We further verified the interaction between LINC00467 and NF-kb-p65 mRNA through RIP experiments. Next, we found that LINC00467 silencing markedly shortened the half-life of NF-kb-p65 mRNA, whereas LINC00467 overexpression markedly increased the half-life of NF-kb-p65 mRNA. Thus, our experiments show that LINC00467 can combine with NF-kb-p65 mRNA and increase its stability. Therefore, our research further improved our understanding of the molecular mechanism by which lncRNAs regulate the NF-κB signaling pathway.

LINC00467 is highly expressed in bladder cancer tissues and can function as an oncogene. LINC00467 can activate the NF-κB signaling pathway to promote the occurrence and development of bladder cancer, and can be used as a potential target for bladder cancer treatment, thus providing new ideas for targeted therapy of bladder cancer.

## Data Availability Statement

The original contributions presented in the study are included in the article/**Supplementary Material**. Further inquiries can be directed to the corresponding author.

## Ethics Statement

The animal study was reviewed and approved by Institutional Review Board of the Third Xiangya Hospital, CSU. Written informed consent was obtained from the individual(s) for the publication of any potentially identifiable images or data included in this article.

## Author Contributions

JX and KC designed the study, analyzed and interpreted the data, and wrote the manuscript. JX, MX, LX, and LD contributed to data acquisition, analysis and interpretation. JX and MX collected clinical database compilation and analysis. MX performed all bioinformatics analysis. JX, MX, LX, LD, and ZW carried out the experiments. ZW and KC provide technical expertise and support. LG conducted the revison work. All authors contributed to the article and approved the submitted version.

## Funding

This work was supported by the National Natural Science Foundation of China (81874137), the Hunan Province Science and Technology Talent Promotion Project (2019TJ-Q10), the Wisdom Accumulation and Talent Cultivation Project of the Third Xiangya Hospital of Central South University (ZC060001), and the Independent Exploration and Innovation Project of Central South University, Grant/Award Number: 2020zzts897.

## Conflict of Interest

The authors declare that the research was conducted in the absence of any commercial or financial relationships that could be construed as a potential conflict of interest.

## Supplementary Material

Supplementary Figure 1
**(A)** The quantification data of protein stability experiments. **(B)** The quantification data of the expression levels of nuclear p-NF-kb-p65 and cytoplasmic NF-kb-p65 after knockdown or overexpression of LINC00467. **(C)** The quantification data of the expression levels of NF-kb-p65 in subcutaneous tumor formation experiments.

Supplementary Figure 2
**(A)** Representative microscopy picture of transfection efficiency. **(B)** The levels of the LINC00467 in four BC cell lines examined by qPCR. **(C)** The levels of the LINC00467 examined by qPCR after knocked-down.
