# Supplementary figures and images for: LINC00467 Promotes Tumor Progression via Regulation of the NF-kb Signal Axis in Bladder Cancer
Source: Front Oncol. 2021 May 28;11:652206. doi: 10.3389/fonc.2021.652206 (PMC8194349; doi:10.3389/fonc.2021.652206)

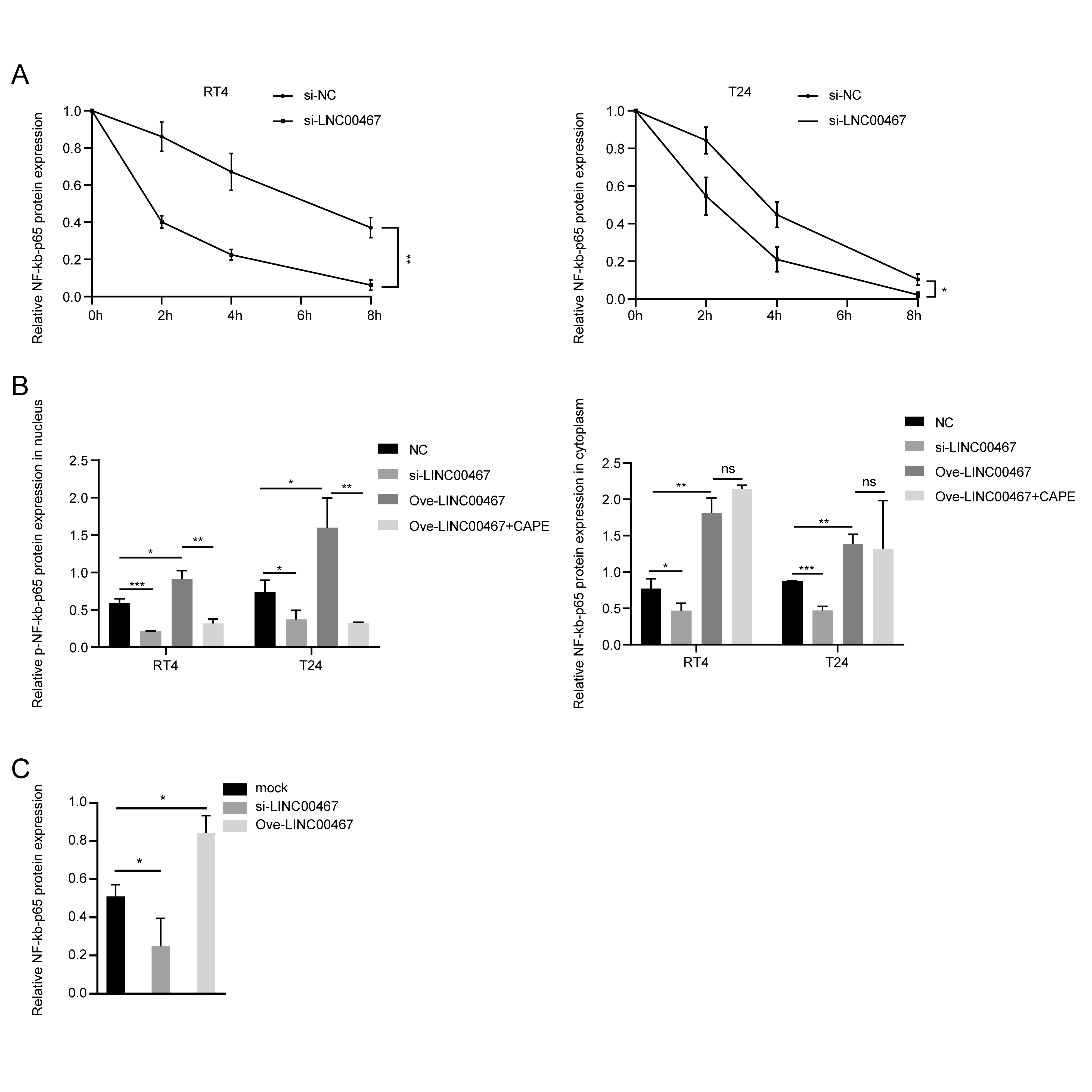

Supplement: Supplementary Figure 1 — (A) The quantification data of protein stability experiments. (B) The quantification data of the expression levels of nuclear p-NF-kb-p65 and cytoplasmic NF-kb-p65 after knockdown or overexpression of LINC00467. (C) The quantification data of the expression levels of NF-kb-p65 in subcutaneous tumor formation experiments. [file Image_1.tif]

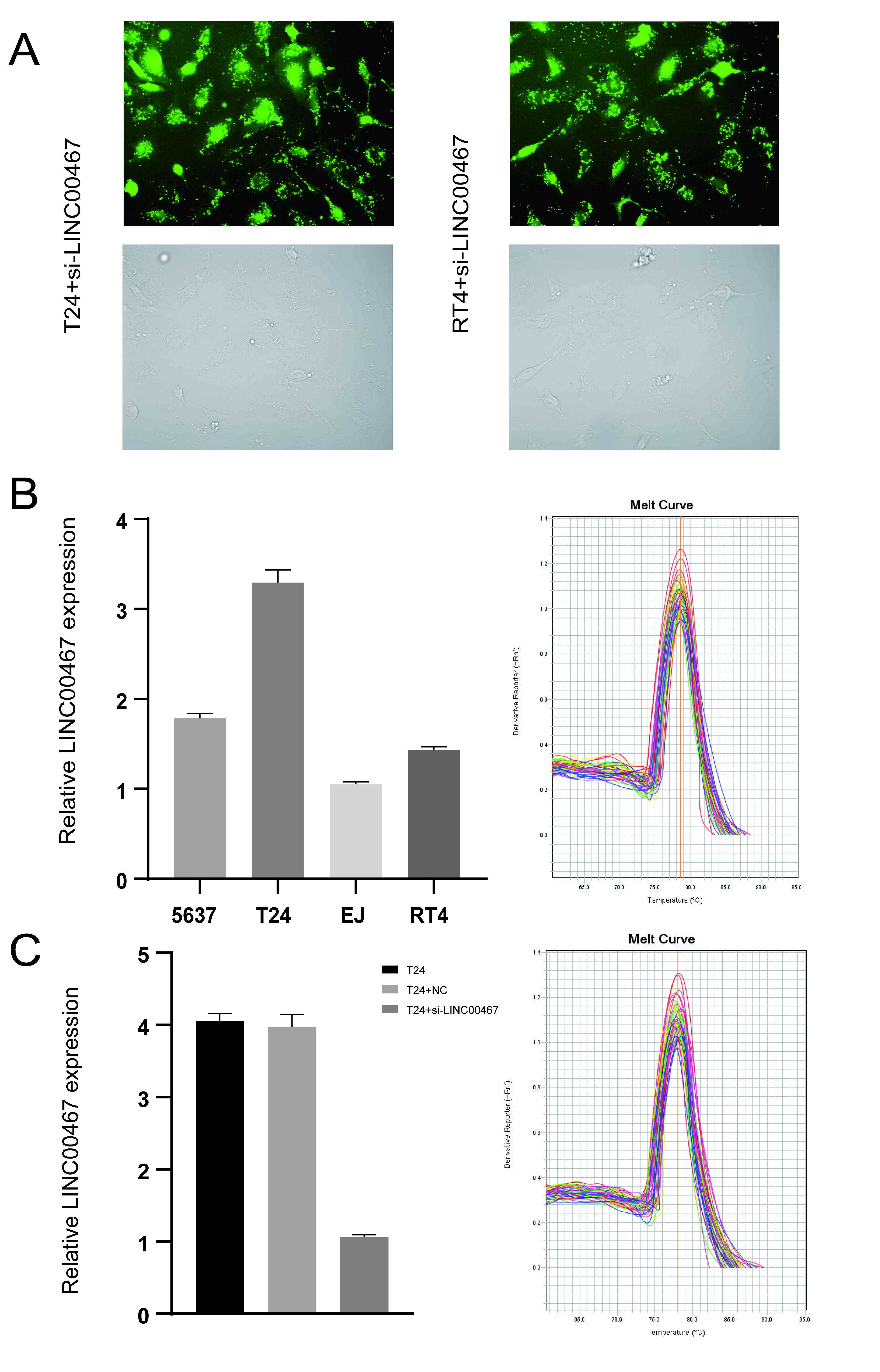

Supplement: Supplementary Figure 2 — (A) Representative microscopy picture of transfection efficiency. (B) The levels of the LINC00467 in four BC cell lines examined by qPCR. (C) The levels of the LINC00467 examined by qPCR after knocked-down. [file Image_2.tif]
